# NaCl-responsive ROS scavenging and energy supply in alkaligrass callus revealed from proteomic analysis

**DOI:** 10.1186/s12864-019-6325-6

**Published:** 2019-12-17

**Authors:** Yongxue Zhang, Yue Zhang, Juanjuan Yu, Heng Zhang, Liyue Wang, Sining Wang, Siyi Guo, Yuchen Miao, Sixue Chen, Ying Li, Shaojun Dai

**Affiliations:** 1 Key Laboratory of Saline-alkali Vegetation Ecology Restoration (Northeast Forestry University), Ministry of Education, College of Life Sciences, Northeast Forestry University, Harbin, 150040 China; 2 Development Center of Plant Germplasm Resources, College of Life Sciences, Shanghai Normal University, Shanghai, 200234 China; 3 College of Life Sciences, Henan Normal University, Xinxiang, 453007 China; 4 Institute of Plant Stress Biology, State Key Laboratory of Cotton Biology, Department of Biology, Henan University, Kaifeng, 455000 China; 5 Department of Biology, Genetics Institute, Plant Molecular and Cellular Biology Program, Interdisciplinary Center for Biotechnology Research, University of Florida, Gainesville, FL 32610 USA

**Keywords:** Salinity response, ROS scavenging, Energy supply, Osmotic homeostasis, Callus, Halophyte alkaligrass, Proteomics

## Abstract

**Background:**

Salinity has obvious effects on plant growth and crop productivity. The salinity-responsive mechanisms have been well-studied in differentiated organs (e.g., leaves, roots and stems), but not in unorganized cells such as callus. High-throughput quantitative proteomics approaches have been used to investigate callus development, somatic embryogenesis, organogenesis, and stress response in numbers of plant species. However, they have not been applied to callus from monocotyledonous halophyte alkaligrass (*Puccinellia tenuifora*).

**Results:**

The alkaligrass callus growth, viability and membrane integrity were perturbed by 50 mM and 150 mM NaCl treatments. Callus cells accumulated the proline, soluble sugar and glycine betaine for the maintenance of osmotic homeostasis. Importantly, the activities of ROS scavenging enzymes (e.g., SOD, APX, POD, GPX, MDHAR and GR) and antioxidants (e.g., ASA, DHA and GSH) were induced by salinity. The abundance patterns of 55 salt-responsive proteins indicate that salt signal transduction, cytoskeleton, ROS scavenging, energy supply, gene expression, protein synthesis and processing, as well as other basic metabolic processes were altered in callus to cope with the stress.

**Conclusions:**

The undifferentiated callus exhibited unique salinity-responsive mechanisms for ROS scavenging and energy supply. Activation of the POD pathway and AsA-GSH cycle was universal in callus and differentiated organs, but salinity-induced SOD pathway and salinity-reduced CAT pathway in callus were different from those in leaves and roots. To cope with salinity, callus mainly relied on glycolysis, but not the TCA cycle, for energy supply.

## Background

Salt stress is a major abiotic threat to plants and has severe effects on agricultural productivity worldwide [1]. Salinity induces ion imbalance, hyperosmotic stress and oxidative damage in plants [2]. Plants have developed complex adaptive mechanisms to cope with the salt stress, such as photosynthetic adjustments, synthesis of osmolytes (e.g., glycine betaine, soluble sugar and proline), and ion homeostasis [3]. In the past years, the salinity-responsive mechanisms in leaves and roots from a number of plant species have been investigated using molecular genetics and different omics strategies [4–9]. In plants, the salt signal perception and transduction, detoxification of reactive oxygen species (ROS), ion uptake/exclusion and compartmentalization, salt-responsive gene expression, protein translation and turnover, cytoskeleton dynamics, cell wall modulation, as well as carbohydrate and energy supply have been investigated in various organs [5, 6]. However, these differentiated organs (e.g., leaves and roots) contain heterogeneous cell types and developmental stages, which may exhibit contrasting sensitivity to salinity. Therefore, it is difficult to determine the cell specific characteristics of salt tolerance when using leaves and roots as materials [10].

Cultured cells are a good model system for investigating cell-specific metabolism because they can be synchronized. Callus obtained by in vitro culture is a group of unorganized cell mass, which has capability to regenerate into a whole plant through somatic embryogenesis and organogenesis. Importantly, callus is an excellent material for genetic transformation in molecular genetics studies. Physiological alterations in calli obtained from sugarcane (*Saccharum officinarum*) [11–13], wheat (*Triticum durum*) [10], rice (*Oryza sativa*) [14, 15], and cotton (*Gossypium hirsutum*) [16] under salinity, osmosis or oxidant conditions were investigated to reveal the stress-responsive mechanisms at cell levels. When being exposed to NaCl stress, sugarcane callus reduced its growth and cell viability, although the cells have the ability to accumulate proline and glycine betaine, and secrete Na^+^ [13]. The growth of sugarcane callus was also decreased under mannitol-induced osmotic stress, likely due to the decreased K^+^ and Ca^2+^ concentrations [12]. The salt-tolerant callus selected from sugarcane cultivar CP65–357 can accumulate more K^+^, proline and soluble sugar, which could facilitate ion and osmotic homeostasis [11]. In general, proline accumulation is an important strategy for osmotic adjustment. However, it has been regarded as an injury symptom rather than an indicator of tolerance in rice callus under salt stress [15]. Among calli from durum wheat (*T. durum*) cultivars with different salt-tolerance capabilities, salt-altered relative growth rate (RGR) and cell viability were correlated, and an induced non-phosphorylating alternative pathway played an important role in salt tolerance [10]. The calli from salt-tolerant wheat cultivar were able to recover after stress relief, and ATP-production was crucial for its growth maintenance [10]. Also, in the callus from NaCl-tolerant cotton, the activities of antioxidant enzymes (e.g., ascorbate peroxidase (APX), catalase (CAT) and glutathione reductase (GR)) were induced, and ROS and nitric oxide played important signaling roles in the course of establishing NaCl tolerance [16]. However, the sophisticated salinity-responsive signaling and metabolic pathways in callus are still unclear.

High-throughput proteomics is a powerful platform for revealing the protein abundance patterns during plant development and environmental responses [17]. Two dimensional electrophoresis (2DE) gel-based and isobaric tags for relative and absolute quantification (iTRAQ) /tandem mass tag (TMT)-based quantitative approaches have been applied to reveal molecular changes during callus development, differentiation and somatic embryogenesis of different plant species, such as sugar cane (*Saccharum* spp.) [18, 19], maize (*Zea mays*) [20–22], rice (*O. sativa*) [23], oil palm (*Elaeis oleifera* × *Elaeis guineensis*) [24], Valencia sweet orange (*Citrus sinensis*) [25], *Cyclamen persicum* [26], *Vanilla planifolia* [27, 28], and lotus (*Nelumbo nucifera* Gaertn. spp. *baijianlian*) [29]. These studies have improved understanding of the molecular regulatory roles of H^+^-pumps (i.e., P H^+^-ATPase, V H^+^-ATPase, and H^+^-PPase), sucrose and pyruvate accumulations, ROS homeostasis, protein ubiquitination, phytohormone and growth regulators (e.g., auxin, cytokinin, abscisic acid and polyamine putrescine) in embryogenic competence acquisition in callus. Importantly, some critical proteins identified in these studies are potential biomarkers for embryogenic competence acquisition, and their functions need to be further investigated [24]. To date, proteomic studies of callus salt tolerance have rarely been reported.

Alkaligrass (*Puccinellia tenuifora*) is a monocotyledonous halophyte with high salinity, alkali and chilling tolerance. It can grow under 600 mM NaCl and 150 mM Na_2_CO_3_ (pH 11.0) for 6 days [30], and can survive chilling stress [31]. Our previous proteomics and physiological studies have reported the salt−/alkali-responsive mechanisms in leaves and roots in response to NaCl (50 mM and 150 mM for 7 days) [32], Na_2_CO_3_ (38 mM and 95 mM for 7 days; 150 mM and 200 mM for 12 h and 24 h) [33–35], and NaHCO_3_ (150 mM, 400 mM and 800 mM for 7 days) [36] stresses. We found alkaligrass accumulated Na^+^, K^+^ and organic acids in vacuoles, as well as proline, betaine and soluble sugar in the protoplasm to maintain osmotic and pH homeostasis in response to salt stress [32, 37]. In these differentiated organs, the fine-tuned mechanisms of signal transduction, ion and osmotic homeostasis, ROS scavenging, transcription and protein synthesis, as well as energy and secondary metabolisms were quite different. However, the salinity-responsive mechanisms in the unorganized callus of alkaligrass have not been reported.

In this study, we investigated the physiological and proteomic characteristics of alkaligrass callus in response to NaCl treatments. The molecular modulations of ROS scavenging, osmotic homeostasis, energy supply, as well as gene expression and protein processing were active in callus under salinity stress. Our results provide new insight into the NaCl response in undifferentiated plant cells, and may have potential applications in the engineering and breeding of salt-tolerant plants.

## Results

### Salinity altered callus growth, viability and membrane integrity

**Fig. 1:**
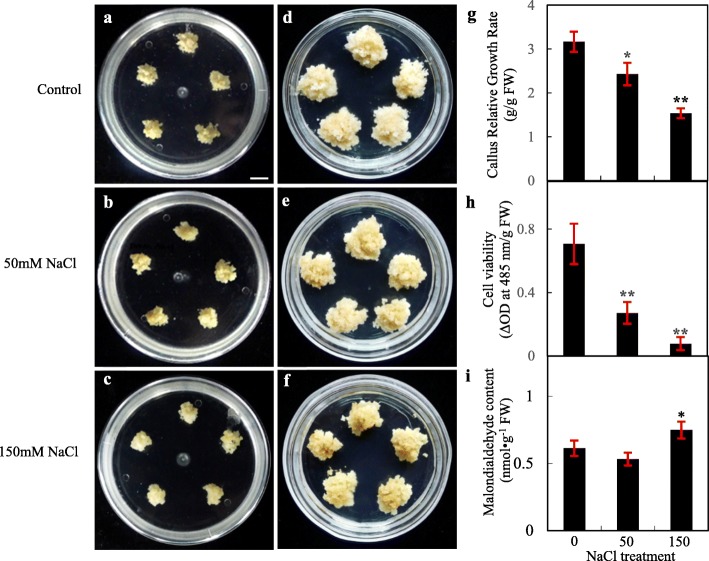
Morphology and growth of alkaligrass calli under NaCl stress. **a**-**f** Morphology of the callus cultured on MS medium. The 45-day-old callus was transferred to MS medium supplemented with 0, 50, and 150 mM NaCl (**a**-**c**), and was cultured for additional 28 days (**d**-**f**). Bar = 1.5 cm. **g** Callus relative growth rate (*n* = 20). **h** Callus cell viability (*n* = 8). **i** Malondialdehyde content (*n* = 3). Values are presented as means ± standard deviation. The values were determined from callus under 0, 50, and 150 mM for 28 days. Significant differences between control and treatments are marked with asterisks (* represents *p* < 0.05, ** represents *p* < 0.01)

After 28 days treatment with NaCl, the callus exhibited obvious phenotypes when compared with control. For example, its growth was decreased with increasing levels of salts. The callus color turned darker under 50 mM NaCl and appeared brown under 150 mM NaCl treatment (Fig. 1a-f). Although the volume of callus mass under control and treatment conditions were significantly increased after 28-day-culture (Fig. 1a-f), their RGR decreased by 1.3-fold and 2.1-fold under 50 mM and 150 mM NaCl, respectively, when compared to control condition (Fig. 1g). Importantly, cell viability was decreased by 62% under 50 mM NaCl and 89% under 150 mM NaCl (Fig. 1h). Furthermore, the membrane integrity of callus cells was affected, as reflected by malondialdehyde (MDA) content. MDA was decreased under 50 mM NaCl, but increased under 150 mM NaCl treatment (Fig. 1i).

### Osmotic homeostasis in callus was disturbed by salt stress

**Fig. 2:**
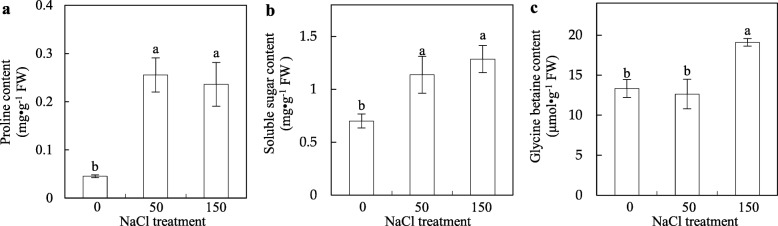
Osmolyte accumulation in the alkaligrass calli under NaCl treatment. **a** Proline content; **b** Soluble sugar content; **c** Glycine betaine content. The values were determined under 0, 50, and 150 mM NaCl and presented as means ± SD (*n* = 3). Different small letters indicate significant difference (*p* < 0.05) among different treatments

To evaluate osmotic adjustment of the callus, the contents of proline, soluble sugar and glycine betaine were determined. The proline contents under 50 mM and 150 mM NaCl treatments were increased by 5.6-fold and 5.2-fold, respectively, compared to the control (Fig. 2a), while the soluble sugar contents were increased by 1.6-fold under 50 mM NaCl and 1.8-fold under 150 mM NaCl treatment (Fig. 2b). The glycine betaine content in callus did not change under 50 mM NaCl, but was significantly increased under 150 mM NaCl (Fig. 2c). These results indicate that the osmotic homeostasis was enhanced by osmolyte synthesis, and the accumulation of proline was marked in NaCl-stressed alkaligrass calli.

### Salt stress-induced ROS levels and antioxidant enzyme activities

**Fig. 3:**
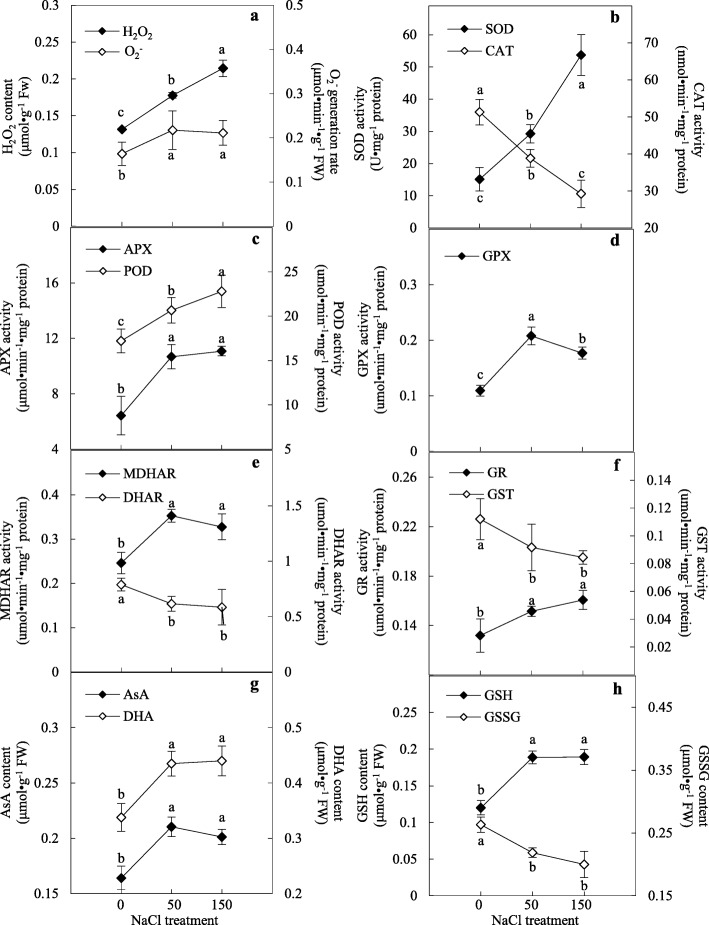
Effect of NaCl on ROS production and antioxidant enzyme activities in the alkaligrass calli. **a** H_2_O_2_ content and O_2_^•-^ generation rate; **b** Activities of superoxide dismutase (SOD) and catalase (CAT); **c** Activities of ascorbate peroxidase (APX) and peroxidase (POD); **d** Glutathione peroxidase (GPX) activity; **e** Activities of monodehydroascorbate reductase (MDHAR) and dehydroascorbate reductase (DHAR); **f** Activities of glutathione reductase (GR) and glutathione S-transferase (GST); **g** Contents of ascorbate (AsA) and dehydroascorbate (DHA); **h** Contents of reduced glutathione (GSH) content and oxidized glutathione (GSSG) content. The values were determined under 0, 50, 150 mM NaCl for 28 days, and were presented as means ± SD (*n* = 3). Different small letters indicate significant difference (*p* < 0.05) among different treatments

To evaluate the ROS levels, H_2_O_2_ content and O_2_^•−^ generation rate in control and NaCl-stressed callus were measured. H_2_O_2_ content and O_2_^•−^ generation were obviously induced by the NaCl treatments (Fig. 3a). This indicates that NaCl treatment triggered oxidative stress in callus cells.

The activities of nine antioxidant enzymes and four antioxidant contents were analyzed (Fig. 3b-h). Among them, superoxide dismutase (SOD) activity was increased by about 1.9-fold under 50 mM NaCl and 3.5-fold under 150 mM NaCl, but the CAT activity was decreased gradually under the two NaCl conditions (Fig. 3b). Conversely, the activities of APX and peroxidase (POD) were both increased under the NaCl treatments (Fig. 3c), and the glutathione peroxidase (GPX) activity was also increased under NaCl treatments (Fig. 3d). Moreover, the activities of monodehydroascorbate reductase (MDHAR), dehydroascorbate reductase (DHAR), and GR in ascorbate-glutathione (AsA-GSH) cycle were perturbed by the NaCl stress. The activities of MDHAR and GR were significantly increased, but the DHAR activity was slightly decreased under the salt stress (Fig. 3e, f). Also, the glutathione S-transferase (GST) activity was decreased under salt stress (Fig. 3f). In addition, the contents of ASA, dehydroascorbate (DHA) and reduced GSH were all increased, concomitant with the decrease of oxidized glutathione (GSSG) under the NaCl treatments (Fig. 3g, h).

### Identification of salt stress-responsive proteins

**Fig. 4:**
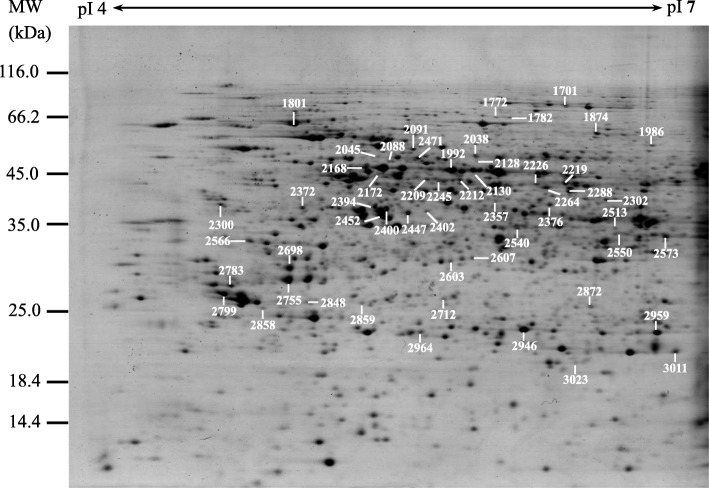
Representative Coomassie Brilliant Blue (CBB)-stained two-dimensional electrophoresis (2DE) gel. Proteins were extracted from the alkaligrass calli under NaCl treatments for 28 days. They were separated on 24 cm IPG strips (pH 4–7 liner gradient) using isoelectric focusing (IEF) in the first dimension, followed by 12.5% SDS-PAGE gels in the second dimension. The numbered gel spots contain the 82 proteins identified by MALDI TOF-TOF mass spectrometry. Please refer to Additional file 1: Figure S1 and Additional file 2: Table S1 for detailed information

**Fig. 5:**
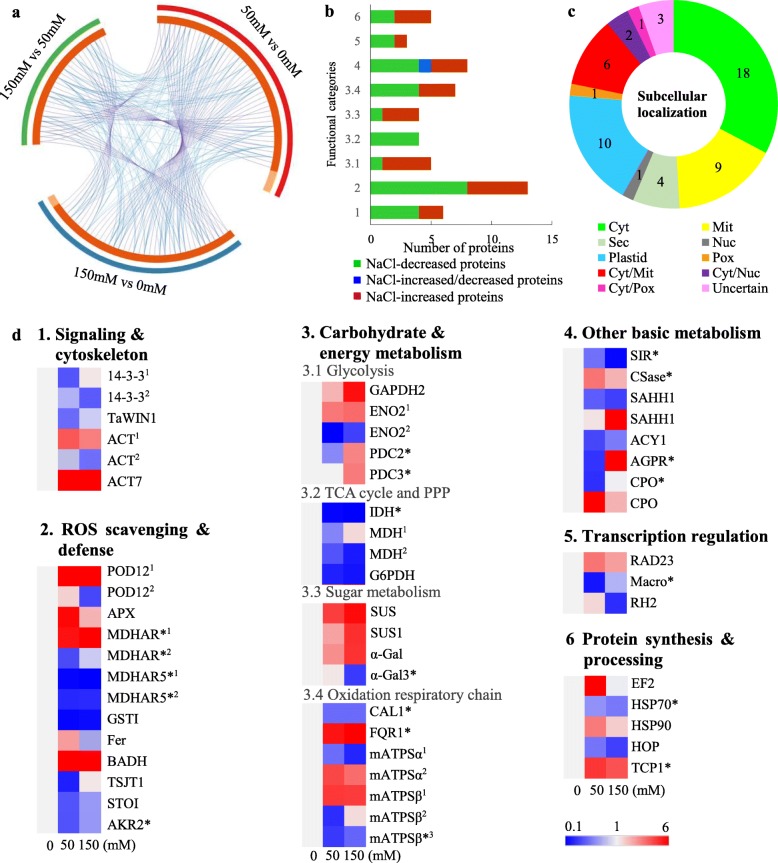
Abundance patterns and subcellular localization of NaCl-responsive proteins in the alkaligrass calli. **a** Overlap between protein lists from three comparison of NaCl treatments. The outer circle represents three comparisons of NaCl treatment conditions (i.e., 50 mM vs 0 mM, 150 mM vs 0 mM, and 150 mM vs 50 mM). The inner circle represents NaCl-responsive protein lists of each treatment conditions, where hits are arranged along the arc. Proteins that hit multiple lists are colored in dark orange, and proteins unique to a list are shown in light orange. The purple and blue curves link the same proteins among three lists. **b** Graphical of the numbers of proteins in various functional categories. The numbers in the y axis are referred in names of function categories in (**d**). **c** Subcellular localization analysis of 55 NaCl-responsive proteins. Subcellular localization categories of proteins predicted by internet tools and knowledge from literatures. The numbers of proteins with different locations are shown in the pie. **d** Abundance patterns of NaCl-responsive proteins in the alkaligrass calli. Three columns represent different treatments of 0, 50, and 150 mM NaCl. The rows represent individual proteins. The increased or decreased proteins are indicated in red or blue, respectively. The color intensity increases with increasing abundant differences, as shown in the scale bar. The scale bar indicates relative protein abundance ratios ranging from 0.1 to 6. The abbreviations of protein name are listed on the right side, and the full names are listed in Additonal file 2: Table S1. The protein names marked with a pentagram (*) were edited according to the functional domain annotations from NCBInr protein database. Abbreviation: 14–3-3^1^, 14–3-3-like protein A; 14–3-3^2^, 14–3-3-like protein GF14–12 isoform X1; α-Gal, Alpha-galactosidase; ACT7, Actin 7 isoform 1; ACY, Aminoacylase-1; AGPR, N-acetyl-gamma-glutamyl-phosphate reductase; AKR2, Aldo-keto reductase 2; APX, Ascorbate peroxidase; BADH, Betaine aldehyde dehydrogenase; CAL1, Carbonic anhydrase-like1; CPO, Oxygen-dependent coproporphyrinogen-III oxidase; Csase, Cysteine synthase; EF2, Elongation factor2; ENO, Enolase; Fer, Ferritin; FQR1, NAD(P)H dehydrogenase (quinone) FQR1-like; G6PDH, 6-phosphogluconate dehydrogenase 1; GAPDH2, Glyceraldehyde-3-phosphate dehydrogenase 2; GSTI, Glutathione S-transferase I; HSP70, Heat shock 70 kDa protein; HSP90, Cytosolic heat shock protein 90.1; HOP, Hsp70-Hsp90 organizing protein 1; IDH, Isocitrate dehydrogenase; Macro, Macro domain-containing protein VPA0103; mATPS, ATP synthase; SIR, Sulfite reductase; MDH, Malate dehydrogenase; MDHAR, Monodehydroascorbate reductase like; PDC, Pyruvate decarboxylase; POD12, Peroxidase12; PPP, Pentose phosphate pathway; RAD23, DNA repair protein RAD23; RH2, DEAD-box ATP-dependent RNA helicase 2; SAHH, S-adenosyl-L-homocysteine hydrolase; STOI, Salt tolerance protein I; SUS, Sucrose synthase; TCP1, T-complex protein 1 subunit theta; TSJT1, Stem-specific protein TSJT 1 like

To investigate protein abundance changes under salt stress, 2DE-based proteomics was employed to separate proteins and analyze their abundance changes. For each callus sample under different NaCl stress conditions, three biological replicates were performed for generating reproducible 2DE results (Fig. 4, Additional file 1). The average spot number on 2DE gels from the three biological replicates was about 1100 in control and treatment samples. Among them, 686, 615 and 657 protein spots were shared in three biological replicates of control, 50 mM and 150 mM NaCl, respectively. In total, 82 protein spots were detected as differentially abundant protein (DAP) spots in calli under NaCl stress (> 1.5-fold and *p* < 0.05). All the DAP spots were excised from 2DE gels, in-gel digested, and subjected to MALDI-TOF MS/MS for protein identification. After Mascot database searching, 55 protein spots were identified to contain a single protein each, and they were taken as NaCl-responsive proteins in alkaligrass calli (Fig. 5, Additional file 2). There were 45 DAPs under 50 mM NaCl and 39 DAPs under 150 mM NaCl when compared with 0 mM NaCl. Among them, 29 DAPs were detected in both NaCl treatment conditions. Four DAPs (i.e., salt tolerance protein 1, aldo-keto reductase 2, cysteine synthase (CSase), and heat shock protein 90.1 (HSP90)) and two DAPs (i.e., actin 2 and heat shock 70 kDa protein (HSP70)) were only identified in callus under 50 mM NaCl and 150 mM NaCl treatment, respectively (Fig. 5a, Additional file 2). Among the 55 NaCl-responsive proteins, 24 were increased and 30 were decreased under one or two treatment conditions, as well as one protein was decreased under 50 mM NaCl, but increased under 150 NaCl treatment (Fig. 5b, Additional file 2).

### Subcellular localization and functional categorization of salt-responsive proteins

The subcellular localization of salt-responsive proteins was predicted based on five Internet tools (i.e., YLoc, LocTree3, Plant-mPLoc, WoLF POSRT and TargetP) and literature. Among them, 18 proteins were predicted to be localized in cytoplasm, ten in plastids, nine in mitochondria, one in nucleus, one in peroxisome, four secreted, and three uncertain. Nine proteins were predicted to be localized in two places, including six in cytoplasm and mitochondria, one in cytoplasm and nucleus, and one in cytoplasm and peroxisome (Fig. 5c, Additional file 4).

Among the 55 DAPs, 20 proteins were originally annotated in the database as unknown proteins, hypothetical proteins, or without annotation. Based on the BLAST alignments and Gene Ontology, 20 proteins were re-annotated (Additional file 3). Subsequently, all the 55 NaCl-responsive proteins were classified into six functional categories, including signaling and cytoskeleton (6 DAPs), ROS scavenging and defense (13 DAPs), carbohydrate and energy metabolism (20 DAPs), other basic metabolism (8 DAPs), transcription regulation (3 DAPs), as well as protein synthesis and processing (5 DAPs) (Fig. 5d).

We identified three NaCl-decreased signaling proteins including two 14–3-3 proteins and a TaWIN1. Thirteen detoxification and oxidative stress-related proteins were identified. They include eight enzymes and five stress-related proteins. Several enzymatic antioxidants (e.g., POD, APX and MDHAR) were increased in abundance under the salt treatments. Other stress-related proteins including ferritin (Fer), betaine aldehyde dehydrogenase (BADH), and stem-specific protein 1 (TSJT1) were increased under NaCl. In addition, AKRs and STO1 were decreased after salt treatments (Fig. 5d, Additional file 2).

The 20 proteins involved in carbohydrate and energy metabolism account for 36.4% of salt-responsive proteins in callus. Several DAPs, such as glyceraldehyde-3-phosphate dehydrogenase (GAPDH), enolase (ENO), and pyruvate decarboxylase (PDC) involved in glycolysis were increased in the salt-treated calli, while other DAPs (e.g., isocitrate dehydrogenase (IDH) and malate dehydrogenase (MDH) in the tricarboxylic acid (TCA) cycle) were decreased in the salt-treated calli. Besides, sucrose synthase (SUS) in sugar metabolism was salt-increased, but 6-phosphogluconate dehydrogenase (G6PDH) in the pentose phosphate pathway (PPP) showed a decrease. In addition, three ATP synthases increased, but two decreased (Fig. 5d).

Three proteins were characterized as transcription-related and five were involved in protein translation and folding. Most of the proteins were increased under salt stress, such as DNA repair protein RAD23, DEAD-box ATP-dependent RNA helicase (RH), elongation factor (EF), HSP70, HSP90, and T-complex protein 1 subunit theta (TCP1). A few protein species were decreased under 150 mM NaCl treatment, such as Macro domain-containing protein, RNA helicase 2, HSP70, and Hsp70-Hsp90 organizing protein 1 (Fig. 5d).

### Protein-protein interaction (PPI) analysis

**Fig. 6:**
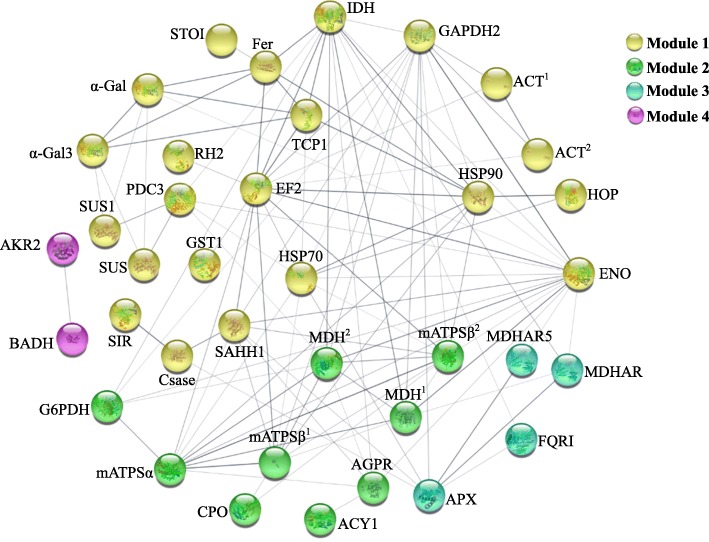
Protein-protein interaction (PPI) network in the alkaligrass calli revealed by STRING analysis. A total of 55 NaCl-responsive proteins represented by 37 unique homologous proteins from *Arabidopsis* are shown in the PPI network. Four modules are presented in different colors. The PPI network is shown in the confidence view generated by STRING database. Stronger associations are represented by thicker lines. Please refer to Fig. 5 and Additional file 5: Table S4 for abbreviations

To evaluate the salt-responsive protein-protein interaction in the callus, a PPI network of NaCl-responsive proteins was visualized using STRING analysis based on homologous proteins in Arabidopsis (Fig. 6, Additional file 5). Among the 55 DAPs, 44 unique homologs were found in Arabidopsis, 37 of which were depicted in the PPI network. Four modules formed tightly connected clusters, and stronger associations were represented by thicker lines in the network (Fig. 6). Module 1 (yellow nodes) contained 22 proteins mainly involved in gene expression, protein synthesis and folding, cytoskeleton dynamics, and glycolysis. The relationship of these proteins indicates that the translation of these proteins involved in glycolysis and cytoskeleton was regulated by EF2, while their processing was mainly dependent on HSP family proteins. Module 2 (green nodes) included nine proteins in TCA cycle, PPP, as well as ATP synthesis and H^+^ supplying, indicating energy supply and H^+^ homeostasis were crucial and interconnected. Module 3 (blue nodes) contained four proteins mainly in charge of ROS scavenging. Module 4 (purple nodes) included two proteins in other basic metabolic processes. In addition, several proteins among four modules also linked with each other. For example, EF2 in module 1 has links with members of ATP synthase (mATP) in module 2, while ENO in module 1 interacted with MDHs, N-acetyl-gamma-glutamyl-phosphate reductase (AGPR) and mATPSα in module 2, as well as MDHARs in module 3. This implies that ATP synthase abundance was modulated by protein translation and diversely-linked energy-supplying pathways, probably being modulated by ROS homeostasis in the callus.

## Discussion

### Salt stress signaling and cytoskeleton dynamics in callus

**Fig. 7:**
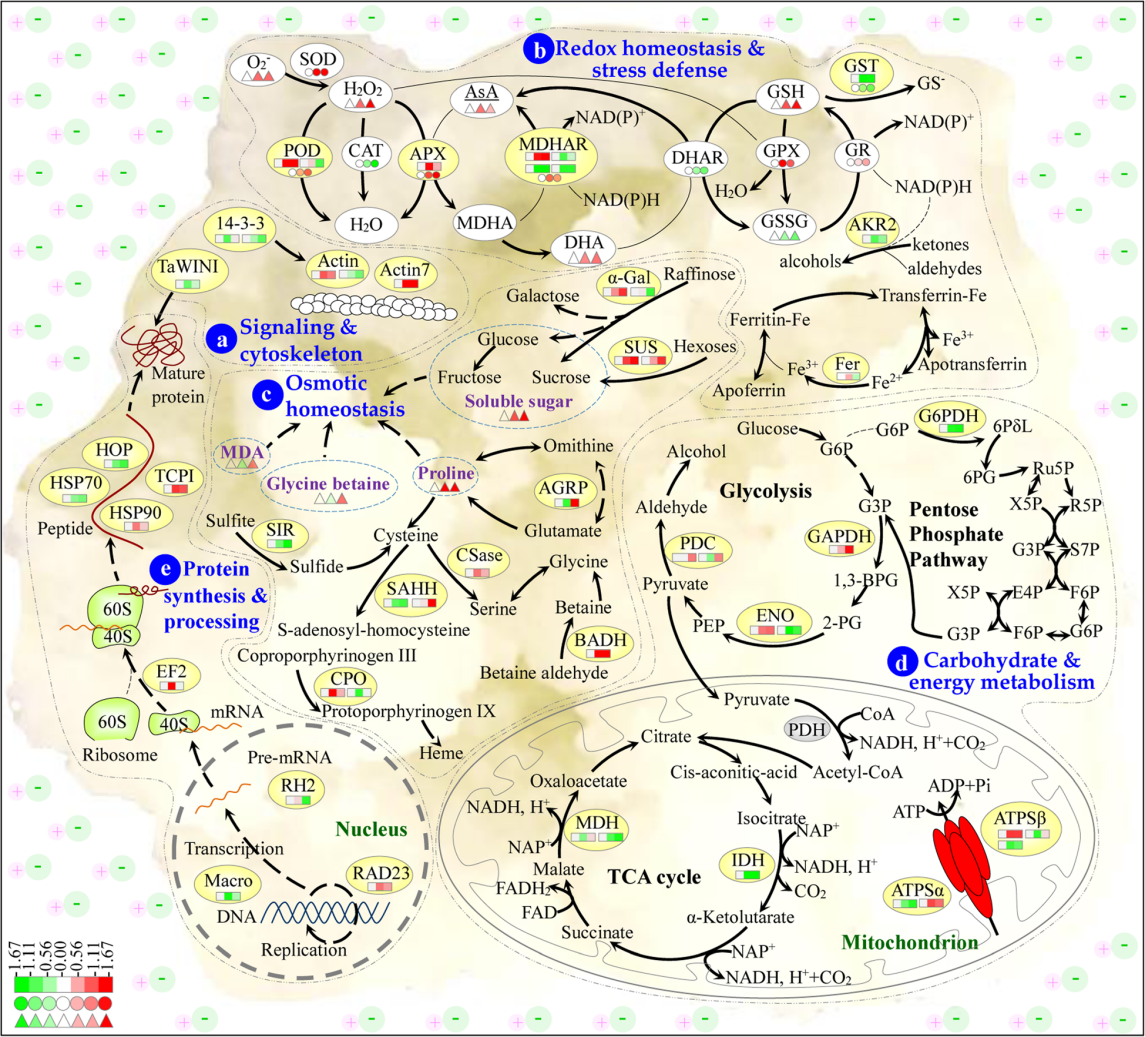
Schematic presentation of systematic salt tolerance mechanisms in the alkaligrass calli. The identified proteins were integrated into subcellular locations and pathways. Relative protein abundances, enzyme activities, and substrate contents in salt treatments compared with control are marked with squares, circles, and triangles white (unchanged), red (increased), and green (decreased), respectively. **a** Signaling and cytoskeleton; **b** Redox homeostasis and stress defense; **c** Osmotic hemostasis; **d** Protein synthesis and processing; **e** Carbohydrate and energy metabolism. Abbreviations: 1,3-BPG, 1,3-bisphosphoglycerate; 2-PG, 2-phosphoglycerate; 6PδL, 6-phosphoglucono-δ-lacton; 6-PG, 6-phosphogluconate; AsA, ascorbic acid; CAT, catalase; DHA, dehydroascorbic; DHAR, dehydroascorbate reductase; E4P, erythrose 4-phosphate; F6P, fructose 6-phosphate; G3P, glycerate 3-phosphate; G6P, glucose 6-phosphate; GPX, glutathione peroxidase; GR, glutathione reductase; GSH, reduced glutathione; GSSG, oxidized glutathione; MDHA, monodehydroascorbate; PEP, phosphoenolpyruvate; POD, peroxidase; R5P, ribose 5-phosphate; Ru5P, ribulose 5-phosphate; S7P, Sedoheptulose 7-phosphate; SOD, Superoxide dismutase; X5P, Xylulose 5-phosphate; Please refer Fig. 5 for abbreviations of proteins identified in this study

Callus development is sensitive to salt stress, and many signaling and metabolic pathways were modulated by the salt treatments. In alkaligrass callus, the signal transduction and cytoskeleton dynamics were altered due to several NaCl-regulated 14–3-3 proteins and actins (Fig. 7a). 14–3-3 proteins as multifunctional proteins regulate diverse downstream target proteins in signal transduction, vesicle trafficking, ion transport, chromatin modulation, and various metabolic pathways in plant response and adaptation to stresses [19]. The abundances of two 14–3-3-like proteins and a TaWIN1 protein were decreased in salt-stressed calli and leaves of alkaligrass [32]. 14–3-3 GF14λ protein was shown to interact with somatic embryogenesis receptor kinase 1 (SERK1) in signal transduction cascade regulating Arabidopsis embryogenic development [38], and to function in the regulation of actin cytoskeleton, cell wall remodeling, and the cell cycle during early stages of somatic embryogenesis of oil palm [39]. Importantly, 14–3-3 proteins were found to trigger programmed cell death in calli from grape [40], barley [41] and tomato [42] in response to biotic stresses, such as infection of *A. tumefaciens* and powdery mildew. In salt stress, whether the decreases of several 14–3-3 proteins help to alleviate salt sensitivity in calli is not known. Their downstream interacting proteins need to be studied toward understanding the 14–3-3 protein-regulated signaling pathways during callus salt response.

Cytoskeletal dynamic changes were revealed from the salt-altered abundance patterns of three actin species (two salt-increased and one decreased). Actin is necessary for callus formation in Arabidopsis [43] and different oil palm species [24, 44]. The depolymerization of the actin cytoskeleton was taken as an important strategy for stress adaptation, triggering the execution of programmed cell death in non-embryogenic callus from maize [21, 45]. It has been reported that Arabidopsis TCP1 helped to facilitate the actin and tubulin protein folding as molecular chaperone to keep the stem cell maintenance [46], suggesting the role of the salinity-induced TCP1 in regulating cytoskeletal dynamics to cope with salt stress.

### Salinity-responsive ROS scavenging pathways in callus

Salt-induced ROS are capable of causing cellular damage by disrupting membrane integrity, degradation of proteins, and inactivation of enzymes [47]. In addition, certain levels of ROS can function to signal cell dedifferentiation and promote somatic embryogenesis in embryogenic callus (EC) [48]. ROS homeostasis in plant calli is fine-tuned [40]. For example, in grape (*Vitis vinifera*) callus, strong APX pathway was triggered in embryogenic callus, whereas CAT pathway was utilized in non-EC [40]. Our study revealed the ROS signal and scavenging networks were sophisticatedly regulated in alkaligrass callus.

The increase of SOD activity contributes to dismutase intracellular O_2_^•-^ to H_2_O_2_ in alkaligrass callus under NaCl stress (Fig. 3b). Similarly, it has been shown that callus from a NaCl-tolerant cotton cultivar exhibited significantly salt-enhanced SOD activity, whereas calli from a NaCl-sensitive cultivar exposed to salt stress showed little change in the SOD activity [49]. Besides, high SOD accumulation was detected in EC compared to non-EC from grapevine (*V. vinifera* L. cv. Cabernet Sauvignon) [40]. This result was different from the NaCl−/Na_2_CO_3_- decreased SOD activities in alkaligrass leaves and roots [32–34]. This result indicates that the undifferentiated salt-tolerant calli from halophyte alkaligrass initially and specifically utilize SOD pathway for ROS homeostasis, when comparing with the salt-sensitive calli and the differentiated organs.

To further maintain homeostasis, the increased H_2_O_2_ is scavenged in diverse pathways. CAT pathway can directly dismutase H_2_O_2_ to H_2_O and O_2_ in peroxisomes. In salinity−/alkali-stressed alkaligrass, CAT activities were decreased in the calli and short-term (12 h and 24 h) salt-treated leaves and roots [34, 35], but increased in long-term (7 days) salt-treated leaves [32, 33]. POD also catalyzes the reduction of H_2_O_2_ using various substrates, such as phenolic compounds, lignin precursors, auxin and secondary metabolites. The NaCl-induced POD activity in the alkaligrass callus implies its crucial role in protecting the callus cells. Similarly, a wide variety of peroxidases were also induced in sugarcane and maize embryogenic calli [19, 22], and peroxidases were very active in ECs from lettuce (*Lactuca sativa*), palm (*Phoenix dactylifera*), and *Medicago truncatula* [50–52]. In addition, the AsA-GSH cycle catalyzed by APX, MDHAR, DHAR and GR participates in the removal of H_2_O_2_, which was implicated in the maintenance of cell wall plasticity and the stimulation of organized cell division [53]. In the alkaligrass callus, the activities of APX, MDHAR and GR were NaCl-increased, but DHAR activity was NaCl-decreased. This result was similar to that in leaves under Na_2_CO_3_-treatmenf for 12 h and 24 h [35]. In addition, the amount of APX and an isoform of MDHAR, as well as the AsA content were all increased in the alkaligrass callus after salt stress. These data suggest that the induced AsA-GSH cycle was critical in regulating the ROS levels in response to salinity stress, and also in maintaining the ability for stimulating cell differentiation upon somatic embryo formation [54].

The increase of GSH and decrease of GSSG are consistent with the induced GPX activity and decreased GST activity in the alkaligrass callus and similar to the changes observed in short-term Na_2_CO_3_-treated roots [34]. The salt-induced CSase in the alkaligrass callus would facilitate the biosynthesis of cysteine, which serves as a precursor for the synthesis of various sulfur-containing metabolites including GSH [25, 55]. It has been reported that cysteine synthase was highly expressed in EC to promote dedifferentiation [54]. Another salinity-increased protein ferritin plays an important role in iron sequestration, preventing the reaction of iron with ROS to cause severe oxidative stress [56]. Clearly, among the complicated ROS pathways in the alkaligrass callus, the NaCl-induced AsA-GSH cycle and POD pathway function to enhance oxidative stress tolerance and maintain cell dedifferentiation, thereby promoting somatic embryo formation [40].

### Diverse osmotic regulation strategies in salt-stressed callus

Under salinity, cell membrane integrity and membrane lipid composition were altered to modulate signal transduction, osmotic homeostasis and cell structure. Cellular MDA contents are often used to evaluate cell membrane damage. In alkaligrass calli under 50 mM and 150 mM NaCl treatments, MDA content was increased (Fig. 1i) but not significantly altered in leaves [32]. However, MDA contents were increased in leaves under Na_2_CO_3_ (38 mM and 95 mM for 7 days) and NaHCO_3_ (150 mM, 400 mM, and 800 mM for 7 days) [33, 36]. This indicates different tissues may respond differently to neutral salt stress and alkali stress.

To cope with osmotic imbalance, diverse compatible osmolytes accumulated in the callus. In this study, the contents of proline, soluble sugar, and glycine betaine were significantly increased (Fig. 2a-c), and BADH involved in glycine betaine biosynthesis was also increased under salt stress (Additional file 2). Proline is not only a compatible osmoticum, but also serves as a storage sink for carbon and nitrogen, stabilizes subcellular structures and buffers cellular redox potential for protecting from oxidative damage under salt stress [57–59]. In leaves of alkaligrass, the proline content was increased under 50 mM and 150 mM NaCl for 7 days [32] or 150 mM, 400 mM, and 800 mM NaHCO_3_ for 7 days [36]. Moreover, proline content was also increased in calli form rice (*O. sativa* cv. KDML 105) [14] and two cultivars of *Medicago sativa* (cv. Yazdi and cv. hamedani) [60] under NaCl stress. In sugarcane (*Saccharum* sp.), proline content is higher in salt-tolerant calli obtained by in vitro selection than in non-selected calli under NaCl stress [11]. Similarly, salt-adapted tobacco calli accumulated more proline than unadapted calli after salt shock [61]. However, in the calli from three sugarcane cultivars with different salt tolerance ability, the salt-resistant cv. R570 accumulated less proline than intermediary salt-resistant cv. NCo310 and salt-sensitive cv. CP59–73 [12]. This perhaps indicates that proline accumulation was merely a stress response, rather than a stress tolerance trait. Similar phenomenon was also reported in calli from tomato [62], rice [63], and *Fraxinus angustifolia* [64] under NaCl stress. All these suggest that the contribution of proline to osmotic adjustment vary in calli derived from different plant species and under different stress conditions. Sometimes, soluble sugars and glycine betaine may play more important roles than proline. For example, soluble sugar content was induced in the alkaligrass calli and leaves under stresses of NaCl [32] or Na_2_CO_3_ [33]. Besides, the salt-tolerant callus of sugarcane accumulated more soluble sugars under NaCl stress [11]. However, the soluble sugars did not accumulate in calli from salt-resistant wheat cv. belikh [10]. In the NaCl-stressed alkaligrass calli, the glycine betaine content was significantly increased. This result was consistent with the results from NaCl-stressed sugarcane calli [13] and the salinity- or alkali treated alkaligrass leaves [32, 35, 36].

### Active glycolysis for energy supply in callus

Calli grow fast and have high cell division rate. A large amount of energy is needed via glycolysis, the TCA cycle, and subsequent energy via the mitochondrial respiratory chain [26]. In the EC of *V. planifolia* and *C. persicum*, glycolytic enzymes were significantly increased due to energy demand of rapid growth and cell division [27, 65]. In this study, eight enzymes involved in glycolysis, PPP, TCA cycle, and other sugar metabolism pathways were altered in the alkaligrass calli under NaCl stress (Figs. 5d and 7d, Additional file 2). Among them, the NaCl-induced GAPDH, ENOs and PDCs suggest that glycolysis was enhanced [66]. Similarly, the protein abundance of GAPDH was increased during *Vanilla planifolia* callus development [27, 28], and the gene expression level of GAPDH was also induced significantly in grape EC [40]. Besides, ENOs were induced in EC relative to non-EC of *V. vinifera* [40], as well as in the developing embryos of spruce (*Picea asperata*) under oxidative stress [67]. These results imply that the increase of glycolysis may promote cellular division and differentiation during callus development and somatic embryogenesis processes [68]. However, the NaCl-decreased IDH and MDH indicate that the TCA cycle was salinity-inhibited in the alkaligrass callus. Consistently, the abundance of various enzymes involved in the generation of acetyl-CoA for the subsequent TCA cycle were lower in EC than in non-EC of maize [20]. In addition, several mitochondria-localized ATP synthase α and β subunits were decreased in the salt-treated alkaligrass calli, consistent with the results obtained in maize and grape [21, 40]. This implies that the energy supply mainly relies on glycolysis, not TCA cycle, because the demand for energy is low in slow growth of EC under salt stress.

Importantly, SUS is a key reversible link of sucrose metabolism in respiration, carbohydrate biosynthesis, and carbohydrate utilization [69]. In this study, two NaCl-induced SUS family proteins could facilitate sucrose flux for energy supply and osmotic homeostasis in the alkaligrass callus. In addition, NaCl-altered alpha-galactosidase (α-Gal) may also help sugar flux for the cell wall expansion in the callus to cope with salinity (Figs. 5d and 7d, Additional file 2).

### Protein synthesis and processing are necessary for callus

Protein synthesis and processing are active during rapid cell reprogramming of somatic embryogenic growth [70, 71]. The proteins involved in protein synthesis and processing accounted for 20% of total proteins in EC and non-EC from saffron (*Crocus sativus*) [70]. The changes to protein profiles observed in our proteomics results imply that transcription, translation, and protein processing in the alkaligrass callus were perturbed by salinity. The altered transcription can be reflected by the NaCl-induced DNA repair protein RAD23, as well as NaCl-decreased Macro domain-containing protein VPA0103 and RH2 [66, 72]. In addition, NaCl-increased EF2 functions in the GTP-dependent ribosomal translocation step during translation elongation. Our PPI analysis showed that EF2 may connect with a number of proteins in energy supply, cytoskeleton, protein folding, and ROS scavenging (Fig. 6). This implies that the enhanced protein synthesis may facilitate diverse pathways in the alkaligrass callus to cope with salinity.

In embryogenic development and callus stress response, removal and refolding of unnecessary and misfolded polypeptides are vital for cell reprogramming [24]. In the alkaligrass calli, we found that salt increased HSP90 and TCP1, as well as salt-decreased HSP70 and Hsp70-Hsp90 organizing protein 1 (HOP). Importantly, our PPI prediction implies that these salt-responsive molecular chaperonins interact with most proteins involved in energy metabolism, protein synthesis, and ROS scavenging (Fig. 6). The accumulation of HSP70 and other HSPs was also found in EC when compared with non-EC from grape [40, 73]. Interestingly, different members of HSP family exhibited diverse abundance patterns during embryo development. For example, HSP60 and HSP101 were typical for the early somatic embryos, while HSP20 and HSP70 marked the late stage of embryogenesis [26]. The assorted chaperon-depended modulations of protein structure indicate that the assembly/structure of the newly-synthesized peptides and stabilization of the mature proteins were dynamically regulated during callus development and stress response [28, 73, 74].

## Conclusion

Investigation of the molecular regulatory mechanisms in callus development and stress response is vital for genetic transformation and plant breeding. Although the NaCl-responsive mechanisms have been reported in leaves and roots, they have not been explored in undifferentiated callus from alkaligrass. In this study, the protein abundance patterns highlight the NaCl-responsive strategies in the alkaligrass calli. Compared with differentiated leaves and roots, the cell membrane of callus cells was more sensitive to salt stress, and significant content changes of the osmolytes (e.g., proline, soluble sugars and glycine betaine) allow synergetic and instantaneous modulation of cellular osmotic homeostasis in salt-stressed calli. Importantly, salinity-induced SOD activities and salinity-decreased CAT activity in the calli were different from those reported in leaves and roots, while the increases of POD activity and AsA-GSH cycle were proposed to be universal strategies in both calli and leaves to cope with salinity and alkali stresses. The halophyte alkaligrass calli mainly relied on glycolysis for energy to cope with salinity, which was similar to the developing EC. In addition, enhanced protein synthesis and folding was crucial for stabilizing diverse proteins involved in energy supply and ROS homeostasis during the callus stress response.

## Methods

### Callus induction and NaCl treatment

Alkaligrass seeds were provided by the Alkali Soil Natural Environment Science Center of Northeast Forestry University, Harbin, China. After removing the seed coat, the mature seeds from alkaligrass (*Puccinelia tenuiflora*) were rinsed in 75% ethanol for 2 min, and washed twice with sterilized water. The seed surface was sterilized in 30% NaClO solution for 5 min, followed by being washed with sterile deionized water 6 times. The surface sterilized seeds were cultured on MS medium, supplemented with 4 mg/L 2,4-dichlorphenoxyacetic acid (2,4-D), 30 g/L sucrose, 4 g/L phytagel, adjusted to pH 5.8. Callus was induced at 25 °C in darkness for 28 days. Proliferating callus were sub-cultured every 28 days on the MS medium. After three rounds of subculturing, the uniform callus was divided into three groups, and treated with 0 mM, 50 mM and 150 mM NaCl in new subculture medium for 28 days. After treatments, callus was harvested and blotted dry briefly on filter paper, and either used fresh or frozen in liquid nitrogen and stored at − 80 °C. For each experiment, at least three biological replicates were collected.

### Callus growth rate and viability determination

The callus RGR was determined on fresh weight (FW) of callus according to the formula: RGR = (final FW - initial FW)/initial FW. The initial FW and final FW were weighted at the 0 day and 28 day of culturing, respectively [12].

The cell viability was determined by a method using 2,3,5-triphenylterazolium chloride (TTC) [10]. Aliquots of 50 mg callus were quickly rinsed in deionized water containing 0.05% Tween 20, and then incubated at 30 °C in darkness containing 5 mL of 0.5% TTC in 50 mM K_2_HPO_4_ (pH 7.0) for 15 h. The samples were filtered through Whatman No. 4 filter paper, rinsed with deionized water, and incubated in 3 mL 94% ethanol at 80 °C for 5 min under gentle agitation to ensure homogenization during the extraction. After centrifugation at 5000×g for 1 min, the extracted formazan was quantified spectrophotometrically at 485 nm. The cell viability is defined as the absorbance measured per gram of fresh tissue (∆OD 485 nm g^− 1^ FW).

### Measurements of MDA, proline, total soluble sugar, and glycine betaine contents

The MDA content in callus was determined by the thiobarbituric acid reaction according to the method of Wang et al. [75]. MDA was extracted from fresh callus in 10% trichloroacetic acid and 0.6% thiobarbituric acid solution under 100 °C for 5 min. The absorbances of the supernatant at 450 nm, 532 nm, and 600 nm were measured as OD450, OD532 and OD600, respectively. The MDA concentration was calculated according to the following equations: C (μmol L^− 1^) = 6.45 x (OD532-OD600) - 0.56 x OD450; MDA concentration (μmol g^− 1^ FW) = C*V/FW (V, volume of total extraction solution; FW, fresh weight of callus).

Proline and total soluble sugar contents were determined using a ninhydrin reaction and an anthrone reagent, respectively, according to the method of Li et al. [76]. The proline was extracted from fresh callus and resolved in 3% sulfosalicylic acid, and then was incubated with the mixture of glacial acetic acid, ninhydrin, and methylbenzene for 2 h. The upper layer of the solution was collected and detected at 520 nm using a spectrophotometer. In addition, the soluble sugar from fresh callus was resolved in deionized water. Then the supernatant was reacted with 2% (w/v) ethyl acetate solution of anthrone and concentrated sulfuric acid, and then measured at 630 nm [76]. The proline and soluble sugar contents were calculated according to the equations from Li et al. [76].

Glycine betaine content was determined using Reinecke salt according to a method from Yu et al. [77]. Briefly, fresh callus was ground to powder in liquid nitrogen, and then incubated in 6 mL 0.375% (w/v) reinecke salt solution for 24 h. The homogenate was centrifuged at 10,000×g at 20 °C for 15 min. The supernatant was filtered, dried, and resuspended in deionized water. After addition of reinecke salt solution, the reaction solution was incubated at 4 °C for 2 h, and then centrifuged at 4000×g at 4 °C for 15 min. The precipitate was washed with ether and 70% acetone. The absorbance was determined at 525 nm using a spectrophotometer.

### ROS measurement and enzyme activity assay

H_2_O_2_ content was determined spectrophotometrically after reaction with potassium iodide [78, 79]. The O_2_^•−^ generation rate was detected using a hydroxylamine oxidization method [77].

The antioxidant enzyme activities were determined according to the methods from Suo et al. [79]. The enzymes were extracted from callus and resolved in phosphate buffer solution (pH 7.8). The supernatant was collected for determination of the activities of SOD, CAT, APX, POD, GPX and GST [79]. For SOD activity detection, the inhibition of the photochemical reduction of nitro blue tetrazolium (NBT) was detected at 560 nm. CAT activity and POD activity were measured by detecting H_2_O_2_ consumption at 240 nm and a guaiacol method at 470 nm, respectively [76]. APX activity and GPX activity were measured by monitoring the absorbance changes at 290 nm and 340 nm, according to the oxidized ascorbate and NADPH, respectively. GST activity was measured at 340 nm, according to the product of CDNB conjugated with GSH absorbed. The activities of MDHAR and DHAR were measured at 340 nm and 265 nm according to the oxidation of NADH and the production of GSSG, respectively [76]. The GR activity was detected at 340 nm as the oxidation of NADPH [76]. Their activities were expressed as the amount of NADH oxidized, GSSG produced, and NADPH oxidized per minute per milligram protein, respectively [80]. For all the enzyme activity assays, protein content was determined using the Bradford method [81].

In addition, the contents of AsA, DHA, GSH, and GSSG were measured by recording the absorbance changes at 525 nm [82].

### Protein sample preparation, 2DE, and protein abundance analysis

Protein extraction was according to the phenol extraction method from Wang et al. [75]. The calli were homogenized in extraction buffer (0.1 M Tris-HCl, pH 8.8, 0.9 M sucrose, 0.4% *β*-mercaptoethanol, 10 mM EDTA) and Tris buffered phenol (pH 8.8). The proteins were precipitated by ammonium acetate in methanol, rinsed with cold acetone, and then dissolved in a lysis buffer (7 M urea, 2 M thiourea, 40 mM DTT, 4% CHAPS, 0.5% IPG buffer pH 4–7). Protein concentration was determined using a Quan-kit (GE Healthcare, Salt Lake City, UT).

Protein samples were separated and visualized on 2D gels according to the method from Wang et al. [75]. The gel images were acquired and analyzed using an ImageScanner III (GE Healthcare) and ImageMaster 2D software (version 5.0) (GE Healthcare). For each sample, three biological replicates were run on 2D gels. More than 1.5-fold change of the average vol% values for protein spot among the treatments and a *p* value < 0.05 were considered to be DAPs [75, 79].

The DAP spots were excised and digested with trypsin according the method of Yu et al. [32]. MS/MS spectra were obtained using an ABI 4800 MALDI TOF/TOF MS (AB SCIEX, Foster City, CA, USA). The mass error was below 30 ppm at both MS and MS/MS mode, and the resolution was 25,000 [75]. The peak list of MS/MS spectra were search against the NCBInr protein database (http://www.ncbi.nlm.nih.gov/protein/) using the online Mascot program (http://www.matrixscience.com). The taxonomic category was Green Plants. The searching parameters were including mass accuracy was 0.3 ppm, the maximum number of missed cleavages was set to one, carbamidomethyl of cysteine as a fixed medication, and oxidation of methionine as a variable modification. The high confident identification of proteins had met the following criteria: the top hits on the database searching report, a probability-based MOWSE score greater than 49 (*p* < 0.05), and more than two peptides matched nearly complete y-ion series and b-ion series.

### Protein classification and hierarchical cluster analysis

To determine their function description, the identified proteins were searched against the NCBI database (https://www.ncbi.nlm.nih.gov/) and UniProt database (http://www.uniprot.org/). Combined BLAST alignments with literature, the identified proteins were classified into different categories. Self-organizing maps analysis of the protein abundances was performed using cluster 3.0 (http://bonsai.hgc.jp/~mdehoon/software/cluster/software.htm) [78].

### Protein subcellular localization prediction and protein-protein interaction (PPI) analysis

The subcellular localization of the identified proteins was predicted using five Internet tools: (1) YLoc (http://abi.inf.uni-tuebingen.de/Services/YLoc/webloc.cgi), confidence score ≥ 0.4; (2) LocTree3 (https://rostlab.org/services/loctree3/), expected accuracy ≥0.8; (3) Plant-mPLoc (http://www.csbio.sjtu.edu.cn/bioinf/plant-multi/); (4) WoLF POSRT (https://wolfpsort.hgc.jp/); (5) TargetP (http://www.cbs.dtu.dk/services/TargetP/), reliability class ≤4 [76]. Only consistent predictions from at least two different tools were accepted. The protein-protein interactions were predicted using the web tool STRING10 (http://string-db.org). The homologs of the different abundance proteins in Arabidopsis were found by sequence BLAST in TAIR database (http://www.arabidopsis.org/Blast/index.jsp). The homologs were subjected to STRING for creating the proteome-scale interaction network [78].

### Statistical analysis

All the results were presented as means ± standard deviation (SD) of at least three biological replicates. The data were subjected to one-way analysis of variance using SPSS 17.0 (SPSS, Chicago, IL, USA). A *p* value smaller than 0.05 was considered to be statistically significant.

## Supplementary information

**Additional file 1: Figure S1.** Three biological replicates of 2DE gels of proteins extracted in from the alkaligrass calli under NaCl treatments.

**Additional file 2: Table S1.** NaCl-responsive proteins identified in the alkaligrass calli using 2DE gel combined with MALDI-TOF MS/MS.

**Additional file 3: Table S2.** Functional annotation of the NaCl-responsive proteins identified in the alkaligrass calli.

**Additional file 4: Table S3.** Subcellular localization prediction of the NaCl-responsive proteins in the alkaligrass calli.

**Additional file 5: Table S4.** Homologs of alkaligrass proteins in Arabidopsis found by sequence BLASTing in TAIR database.

## Data Availability

The data sets supporting the results of this article are included within the article and its additional files.

## Abbreviations

2DE: Two-dimensional electrophoresis
AKR2: Aldo-keto reductase 2
APX: Ascorbate peroxidase
ASA: Ascorbate
BADH: Betaine aldehyde dehydrogenase
CAT: Catalase
DAP: Differentially abundant protein
DHA: Dehydroascorbate
DHAR: Dehydroascorbate reductase
EC: Embryogenic callus
EF: Elongation factor
ENO: Enolase
Fer: Ferritin
FW: Fresh weight
G6PDH: 6-phosphogluconate dehydrogenase
GAPDH: Glyceraldehyde-3-phosphate dehydrogenase
GPX: Glutathione peroxidase
GR: Glutathione reductase
GSH: Glutathione
GSSG: Oxidized glutathione
GST: Glutathione S-transferase
HOP: Hsp70-Hsp90 organizing protein
HSP70: Heat shock 70 kDa protein
HSP90: Heat shock protein 90.1
IDH: Isocitrate dehydrogenase
mATP: ATP synthase
MDA: Malondialdehyde
MDH: Malate dehydrogenase
MDHAR: Monodehydroascorbate reductase
PDC: Pyruvate decarboxylase
POD: Peroxidase
PPI: Protein-protein interaction
PPP: Pentose phosphate pathway
RGR: Relative growth rate
RH: RNA helicase
ROS: Reactive oxygen species
SOD: Superoxide dismutase
STO1: Salt tolerance protein 1
SUS: Sucrose synthase
TCA: Tricarboxylic acid
TCP1: T-complex protein 1
TSJT1: Stem-specific protein 1
