# Supplementary material for: NaCl-responsive ROS scavenging and energy supply in alkaligrass callus revealed from proteomic analysis
Source: BMC Genomics. 2019 Dec 17;20:990. doi: 10.1186/s12864-019-6325-6 (PMC6918623; doi:10.1186/s12864-019-6325-6)
Supplement: Supplementary file 1 — Additional file 1: Figure S1. Three biological replicates of 2DE gels of proteins extracted in from the alkaligrass calli under NaCl treatments. [file 12864_2019_6325_MOESM1_ESM.docx]

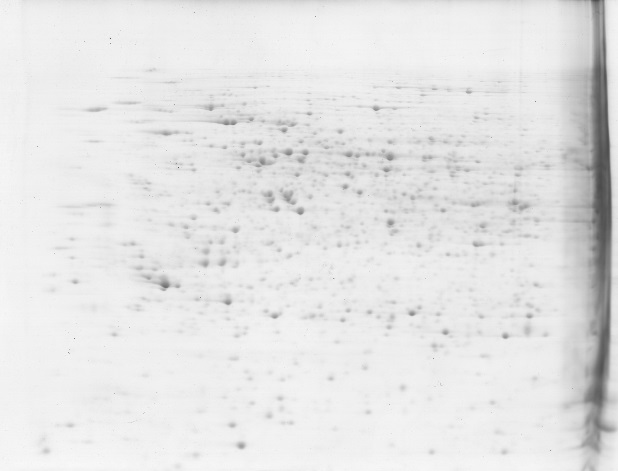


**a1**


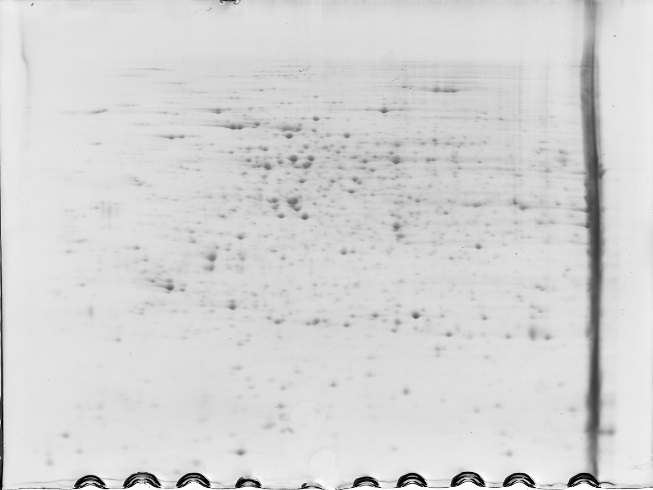


**b1**


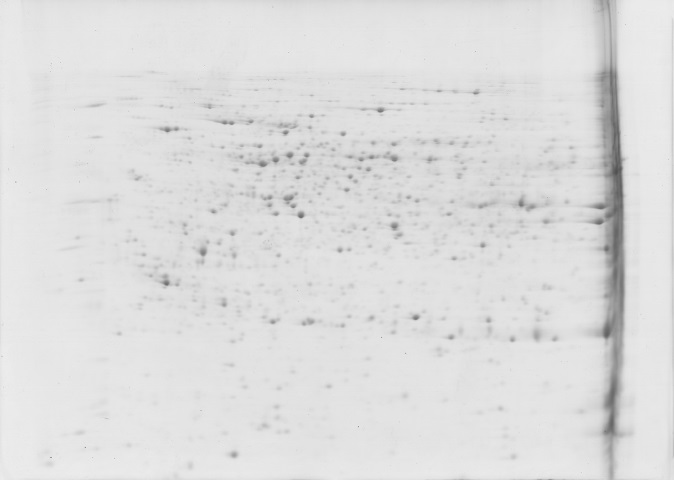


**c1**

pI 4

7

pI 4

7

MW (KDa)

116.0

66.2

14.4

45.0

18.4

35.0

25.0

pI 4

7


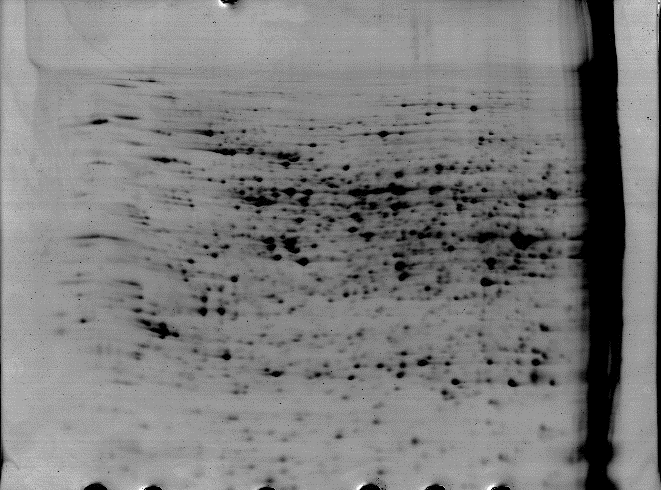


**a2**


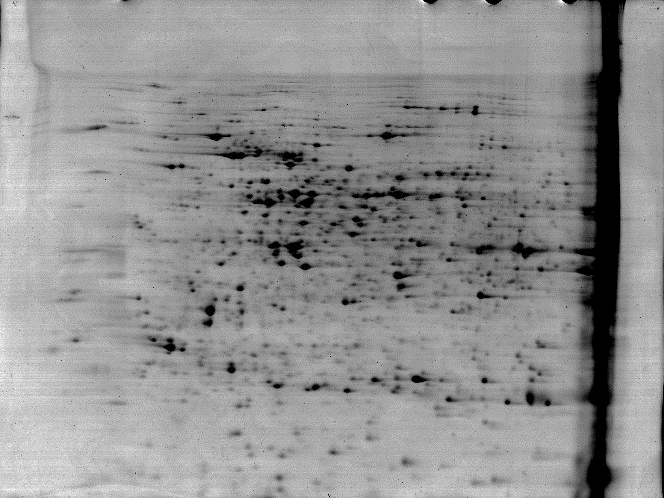


**b2**


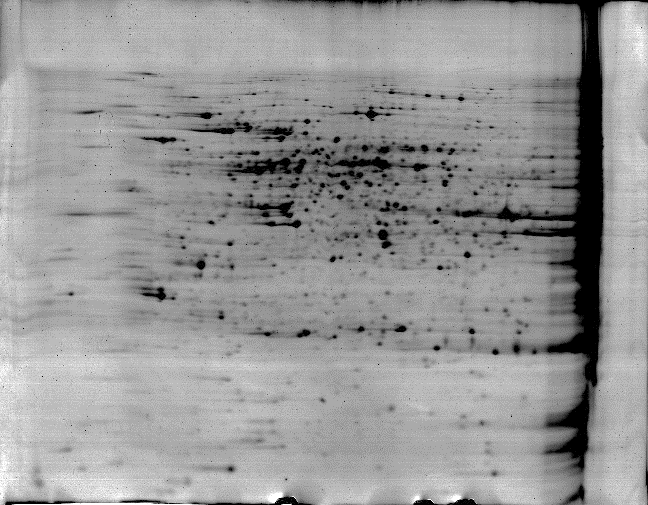


**c2**

pI 4

7

pI 4

7

MW (KDa)

116.0

66.2

14.4

45.0

18.4

35.0

25.0

pI 4

7


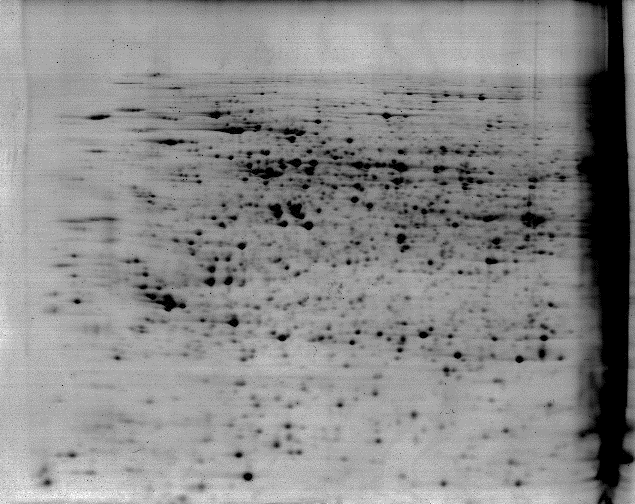


**a3**


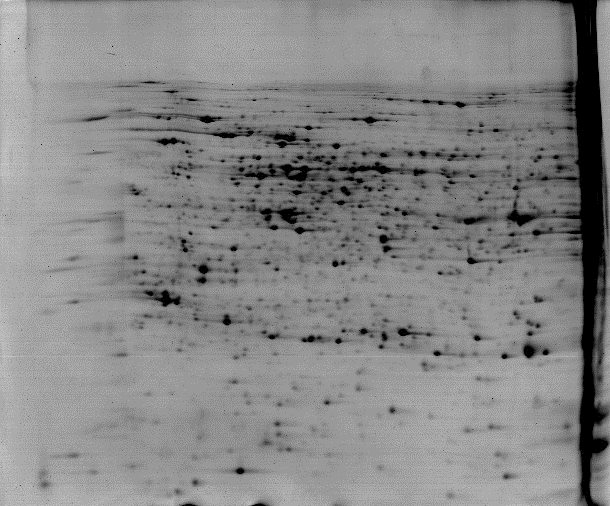


**b3**


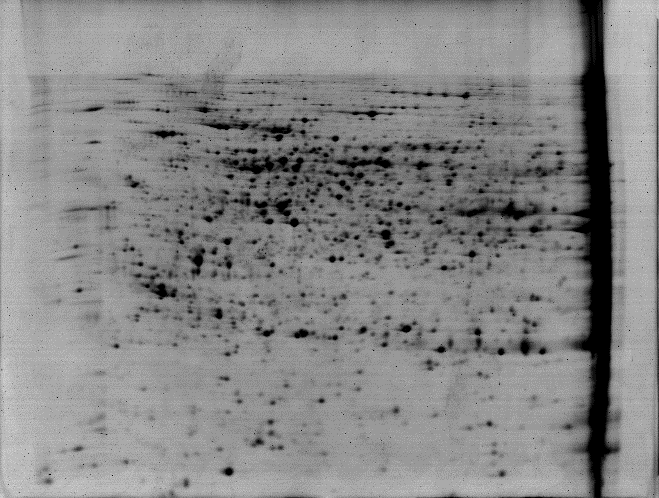


**c3**

pI 4

7

pI 4

7

MW (KDa)

116.0

66.2

14.4

45.0

18.4

35.0

25.0

pI 4

7

**Supplemental Fig. S1.** Three biological replicates of two dimensional electrophoresis (2DE) gels of proteins extracted in alkaligrass calli under NaCl treatments. **a1-a3** 2DE gels of samples under 0 mM NaCl; **b1-b3** 2DE gels of samples under 50 mM NaCl; **c1-c3** 2DE gels of samples under 150 mM NaCl. Proteins were separated on 24 cm linear gradient IPG strips (pH 4–7) using isoelectric focusing (IEF) in the first dimension, followed by 12.5% SDS-PAGE gels in the second dimension. The 2DE gel was stained with Coomassie Brilliant Blue. Molecular weight (MW) in kilodaltons (kDa) and *p*I of proteins are indicated on the left and top of the gels, respectively.
